# Associated risk factors with disease severity and antiviral drug therapy in patients with COVID-19

**DOI:** 10.1186/s12879-021-06282-6

**Published:** 2021-06-10

**Authors:** Xiaowei Gong, Shiwei Kang, Xianfeng Guo, Yan Li, Haixiang Gao, Yadong Yuan

**Affiliations:** 1 Department of Respiratory and Critical Care Medicine, The Second Hospital of Hebei Medical University, Shijiazhuang, 050000 China; 2 Department of orthopedics, No. 7 Hospital of Wuhan, Wuhan, China; 3 Department of Respiratory Medicine, Hebei General Hospital, Shijiazhuang, China

**Keywords:** COVID-19, SARS-CoV-2, Disease severity, Associated factors, Antiviral drug

## Abstract

**Background:**

Due to the latent onset of novel coronavirus disease 2019 (COVID-19), it is important to identify patients with increased probabilities for disease progression early in order to implement timely medical strategies. This study aimed to identify the factors associated with increased COVID-19 severity and evaluate the current antiviral drugs, especially in severe patients.

**Methods:**

This was a retrospective observational study performed at the No. 7 Hospital of Wuhan (Wuhan, China) with hospitalized patients confirmed with COVID-19 from January 11 to March 13, 2020. Multivariable logistic regression analysis was used to identify the associated factors of severe COVID. Treatments of antivirus drugs were collected and evaluated.

**Results:**

Of the 550 patients, 292 (53.1%) were female and 277 (50.4%) were > 60 years old. The most common symptom was fever (*n* = 372, 67.7%), followed by dry cough (*n* = 257, 46.7%), and dyspnea (*n* = 237, 43.1%), and fatigue (*n* = 224, 40.7%). Among the severe patients, 20.2% required invasive ventilator support and 18.0% required non-invasive ventilator. The identified risk factors for severe cases were: age ≥ 60 years (odds ratio (OR) =3.02, 95% confidence interval (CI): 1.13–8.08, *P* = 0.028), D-dimer > 0.243 μg/ml (OR = 2.734, 95%CI: 1.012–7.387, *P* = 0.047), and low oxygenation index (OR = 0.984, 95%CI: 0.980–0.989, *P* < 0.001). In severe cases, the benefits (relief of clinical symptoms, clinical outcome, and discharge rate) of arbidol alone was 73.3%, which was better than ribavirin (7/17, 41.2%, *P* = 0.029).

**Conclusions:**

Age > 60 years, D-dimer > 0.243 μg/ml, and lower oxygenation index were associated with severe COVID-19. Arbidol might provide more clinical benefits in treating patients with severe COVID-19 compared with ribavirin.

## Background

A novel member of the coronavirus family, severe acute respiratory syndrome coronavirus 2 (SARS-CoV-2), has infected more than 42 million people in the world as of October 27, 2020, since it was first identified in December 2019, causing over 1.1 million deaths [1–7]. SARS-CoV-2 is found on all continents and in nearly all countries [6]. On March 11, 2020, the World Health Organization (WHO) announced that the coronavirus disease 2019 (COVID-19) had become a pandemic [8]. Currently, the domestic epidemic in China has been largely controlled after a painful nation-wide war against COVID-19. Unfortunately, this epidemic occurred when our knowledge about similar previous viruses, the acute respiratory syndrome coronavirus (SARS-CoV) and Middle East respiratory syndrome coronavirus (MERS-CoV), was still limited. This lack of knowledge severely limited the response against the virus and its spread.

Indeed, the SARS-CoV-2 exhibits 79.5% homology with SARS-CoV, and patients infected with either of them have similar symptoms, but SARS-CoV-2 is more contagious than SARS-CoV, as shown by an increased reproduction number (R_0_) [9]. Complicating screening, control, and management, the clinical symptoms of COVID-19 are non-specific (fever, cough, and shortness of breath) and are shared by a number of respiratory infections. In addition, many patients are asymptomatic, most of those with symptoms have a good prognosis, and about 20% of symptomatic patients may experience disease progression and reach a critical condition [10]. Such patients will quickly progress to acute respiratory distress syndrome (ARDS), respiratory failure, multiple organ failure (MODS), or even death [1–5]. Suspected risk factors for severe COVID-19 include age > 65 years, residence in long-term care facilities, and underlying conditions such as chronic lung disease, a serious heart condition, severe obesity, diabetes, chronic kidney disease, liver disease, and immunocompromising conditions [11].

The virus is still spreading relentlessly and exponentially around the world and creating an enormous threat to global health, with the fear of second and later waves [6]. Unfortunately, no specific therapeutic drugs are yet available. Currently, the treatment of severe and critical cases involves support treatments aiming at maintaining oxygenation and controlling inflammation and coagulation [12, 13]. Drugs such as hydroxychloroquine have been tried against COVID-19 but finally was not proven effective [14, 15]. Antiviral drugs might reduce infection duration and time to symptom resolution [16–19]. A study showed that patients receiving remdesivir recovered 5 days faster than those receiving placebo, while 86% of severe patients recovered an average of 7 days faster [20]. Favipiravir was not associated with the viral clearance rate but with a reduced time to defervescence [21]. Similar benefits were suggested for interleukin (IL)-6 inhibitors [22, 23]. Nevertheless, no drug is universally recognized to be effective against COVID-19 [22].

Besides, COVID-19 could elapse as much as 2–3 weeks between virus exposure and symptom onset, and could result in severe or even fatal complications [1–5]. The early identification of patients with an increased likelihood of disease progression is important in order to implement timely medical strategies and to adjust them according to the evolving conditions, especially in the context of the exhausted healthcare systems around the world. Given the limited data on the risk factors for severe patients and effectiveness of antiviral drugs, we determined to identify the clinical factors associated with severe COVID-19 and evaluate the current antiviral drugs, especially in patients with severe COVID-19.

## Methods

### Study design and patients

This single-center, retrospective, observational study was performed at the No. 7 Hospital of Wuhan (Wuhan, China), which is a designated hospital to treat patients with COVID-19. The medical team from the Second Hospital of Hebei Medical University was appointed by the government to provide medical assistance to the No. 7 Hospital of Wuhan during the outbreak. All hospitalized patients diagnosed with “viral pneumonia” from January 11 to March 13, 2020, were preliminarily included in this study. Patients confirmed with COVID-19 were then enrolled in the study. The diagnosis was made following the Chinese COVID-19 management guideline (versions 3 to 7) [12]. Patients with atypical clinical symptoms or chest radiology changes combined with negative SARS-CoV-2 RNA test results were excluded from this study. The study was approved by the Institutional Ethics Board of the No. 7 Hospital of Wuhan and the Second Hospital of Hebei Medical University (Code: 2020-R016). The need for individual consent was waived due to the non-interventional and retrospective nature of this study.

### Data collection

The patients’ electronic medical records, which had been archived onto a local server, including epidemiology parameters, clinical presentation, laboratory results, imaging characteristics, treatments, and disease outcomes, were collected by a group of designated physicians who accepted sufficient training. Several important time points were also analyzed, including disease onset, time from disease onset to dyspnea, time for SARS-CoV-2 RNA to be no longer detectable in patients with positive RNA results upon hospital admission, and average hospital stay. For patients who required mechanical ventilation, time from disease onset to ARDS and time to mechanical ventilation were analyzed. All data were checked by two physicians (XWG and SWK). Major disagreement between two physicians was resolved by consultation with a third reviewer (YDY). Any missing or uncertain records were obtained and clarified through direct communication with attending doctors and their families.

### Definition

The exposure history denote that a person in close contact with diagnosed patients or recent visiting of COVID-19 designated hospitals within 2 weeks before the onset of respiratory symptoms. Fever was defined as axillary temperature of at least 37.3 °C. Acute myocardial injury was diagnosed if serum levels of cardiac biomarkers (eg, troponin T) were above the 99th percentile upper reference limit, or if new abnormalities were shown in electrocardiography and echocardiography [1]. Secondary infection was diagnosed after the occurrence of hospital-acquired pneumonia or bacteremia, plus a positive result of new pathogen culture from the blood and lower respiratory tract specimens (including sputum, bronchoalveolar lavage fluid or tracheal aspirate) after admission [24]. Acute respiratory distress syndrome (ARDS) was diagnosed according to the Berlin Definition [25]. Acute kidney injury was diagnosed according to the KDIGO clinical practice [26]. Shock and organ failure was defined in accordance with the Chinese COVID-19 management guideline [12]. DIC was diagnosed according to ISTH criteria [27].

Disease onset was defined as the time of patients starting to present symptoms. The severe cases were identified according to the Chinese COVID-19 management guideline (versions 3 to 7) [12]. Disease progression was identified and classified when the patients had one of the following criteria: 1) respiratory distress with respiratory frequency ≥ 30/min; 2) pulse oximeter oxygen saturation ≤ 93% at rest; and 3) oxygenation index (artery partial pressure of oxygen/inspired oxygen fraction, PaO2/FiO2) ≤300 mmHg [12]. Disease improvement was defined as: patients’ situation remained unchanged; severe cases changed to non-severe cases; and patients were permitted for discharge. The discharge criteria were: body temperature returned to normal and maintained for more than three consecutive days; significantly improved respiratory symptoms; a significant improvement on imaging and a negative result on RNA tests with two consecutive sputum samples or nasopharyngeal swabs or other respiratory samples (at least 24 h between each sampling) [12].

### Laboratory tests

The pharynx swabs of suspected patients were collected and transported to the clinical laboratory of Zhongnan Hospital of Wuhan University for RNA detection following strict standard procedures. The presence of SARS-CoV-2 in pharynx swabs was detected by real-time RT-PCR. The detailed analysis and detection processes can be found in a previous study [3]. Laboratory tests and radiologic assessments, including chest X-ray or computed tomography (CT), were performed on the basis of the state of illness.

### Treatment

Treatment was provided according to the Chinese COVID-19 management guideline (versions 3 to 7) [12], combined with the clinical characteristics of the patients and the actual situation of the medical resources of No. 7 Hospital of Wuhan during the epidemic. Patients with a mild condition were given general support like resting in bed, supportive treatment, antiviral treatment, and antibiotics if necessary. Severe patients were given respiratory and other organs support treatment on an individualized basis. We comprehensively evaluated the treatment effect of the patients by closely observing their clinical condition change and disease outcome.

### Statistical analysis

Categorical variables were presented as frequencies and percentages and analyzed using the chi-square test or Fisher’s exact test, as appropriate. Continuous variables were presented as mean ± standard deviations or medians (interquartile range (IQR)) according to the results of the Kolmogorov-Smirnov test and analyzed using Student’s t-test or the Mann-Whitney U-test, according to the distribution. Variables were first screened with univariable logistic regression; variables with *P*-values < 0.05 for association with severe COVID-19 were included in a multivariable logistic regression analysis. In univariable analysis, we chose a total of 19 variables which had been commonly observed in severe or non-surviving patients [1–5]. Variables were excluded if their *P*-values > 0.05, if their accuracy could not be confirmed (symptom, which was self-reported), if they were unavailable under emergency circumstances (radiographic findings, troponin T, glomerular filtration rate), if the sample size was relatively small (diabetes, heart disease), if they might be related to other variables (sex, leucocytes, procalcitonin). Besides, considering the earlier analysis of the total number of deaths (*n* = 52) in this study and to avoid model overfitting, the six variables with the strongest association were selected for the multivariable logistic regression analysis on the basis of previous findings and clinical constraints. All statistical analysis was performed using SPSS 22.0 (IBM, Armonk, NY, USA). Two-sided (except for the chi-square test) *P*-values < 0.05 were considered statistically significant.

### Patient and public involvement

This was a retrospective case series study, and no patients were involved in the study design or in setting the research questions or the outcome measures directly. No patients were asked to advise on the interpretation or writing up of results.

## Results

### Demographic and clinical characteristics

All hospitalized patients (*n* = 644) diagnosed with “viral pneumonia” from January 11 to March 13, 2020, were screened for inclusion. Finally, 550 patients diagnosed with COVID-19 (including 422 cases positive for SARS-CoV-2 RNA and 128 cases clinically diagnosed but with negative RNA tests) were included. Table 1 presents the characteristics of the patients. Among all patients, 292 (53.1%) patients were female, and 258 (46.9%) were male. Most patients were > 60 years; 277 (50.4%) and 342 (62.2%) reported no history of exposure to COVID-19. Hypertension (*n* = 184, 33.5%), diabetes (*n* = 77, 14.0%), cardiovascular disease (*n* = 56, 10.2%), and malignancy (*n* = 23, 4.2%) were the most frequent comorbidities. Ultimately, 178 patients progressed to a severe condition (32.4%), and 52 died (9.5%).

**Table 1:** Demographics and comorbidities of patients with COVID-19

Variables	All Patients (*n* = 550)	Severe (*n* = 178)	Non-severe (*n* = 372)	*p* value
Age, years, n (%)				
< 44	103 (18.7%)	13 (7.3%)	90 (24.2%)	< 0.001
45–59	170 (30.9%)	36 (20.2%)	134 (36.0%)	< 0.001
≥ 60	277 (50.4%)	129 (72.5%)	148 (39.8%)	< 0.001
Sex, n (%)				
Female	292 (53.1%)	74 (41.6%)	218 (58.6%)	< 0.001
Male	258 (46.9%)	104 (58.4%)	154 (41.4%)	< 0.001
Source of transmission, n (%)				
None	342 (62.2%)	144 (80.9%)	198 (53.2%)	< 0.001
Contact history with diagnosed patients	170 (30.9%)	22 (12.4%)	148 (39.8%)	< 0.001
Recent visit of COVID-19 designated hospitals	38 (6.9%)	12 (6.7%)	26 (7.0%)	< 0.001
Comorbidity, n (%)				
Hypertension	184 (33.5%)	81 (45.5%)	103 (27.7%)	< 0.001
Diabetes	77 (14.0%)	37 (20.8%)	40 (10.8%)	0.002
Cardiovascular disease	56 (10.2%)	26 (14.6%)	30 (8.1%)	0.018
Malignancy	23 (4.2%)	13 (7.3%)	10 (2.7%)	0.011
Cerebrovascular disease	20 (3.6%)	10 (5.6%)	10 (2.7%)	0.086
Chronic pulmonary disease	18 (3.3%)	7 (3.9%)	11 (3.0%)	0.547
Chronic liver disease	15 (2.7%)	4 (2.2%)	11 (3.0%)	0.843
Hyperlipidemia	10 (1.8%)	5 (2.8%)	5 (1.3%)	0.229

Data are shown as median (IQR) or n (%). *ARDS* Acute respiratory distress syndrome, *CMV* Cytomegalovirus. *EBV* Epstein-Barr virus

The most common symptom upon diagnosis was fever (*n* = 372, 67.6%), most frequently between 38 and 39 °C (*n* = 204, 37.1%) (Table 2). It is important to point out that a significant portion of patients (*n* = 178, 32.3%) did not have fever at diagnosis. The remaining common symptoms were dry cough (*n* = 257, 46.7%), dyspnea (*n* = 237, 43.1%), fatigue (*n* = 224, 40.7%), sputum production (*n* = 169, 30.7%), and abdominal pain/diarrhea (*n* = 75, 13.6%). Most patients (*n* = 393, 71.5%) presented with more than one symptom, but only 130 (23.6%) showed the classical triple signs of COVID-19 (fever, cough, and dyspnea).

**Table 2:** Clinical characteristics, radiographic, and etiology of patients with COVID-19

Variables	All Patients (*n* = 550)	Severe (*n* = 178)	Non-severe (*n* = 372)	*p* value
Surgical history, n (%)	90 (16.4%)	24 (13.5%)	66 (17.7%)	0.207
Signs and symptoms, n (%)				
Fever				
< 37.3 °C	178 (32.3%)	21 (11.8%)	157 (42.3%)	< 0.001
37.3–38.0 °C	117 (21.3%)	35 (19.7%)	82 (22.0%)	< 0.001
38.0–39.0 °C	204 (37.1%)	93 (52.2%)	111 (29.8%)	< 0.001
> 39.0 °C	51 (9.3%)	29 (16.3%)	22 (5.9%)	< 0.001
Dry cough	257 (46.7%)	90 (50.6%)	167 (44.9%)	0.212
Dyspnea	237 (43.1%)	119 (66.9%)	118 (31.7%)	< 0.001
Fatigue	224 (40.7%)	91 (51.1%)	133 (35.8%)	0.001
Sputum production	169 (30.7%)	70 (39.3%)	99 (26.6%)	0.002
Chill	123 (22.4%)	52 (29.2%)	71 (19.1%)	0.008
Stomachache/Diarrhea	75 (13.6%)	29 (16.3%)	46 (12.4%)	0.209
Nausea/Vomit	70 (12.7%)	22 (12.4%)	48 (12.9%)	0.858
Myalgia	57 (10.4%)	18 (10.1%)	39 (10.5%)	0.894
Sore throat	46 (8.8%)	12 (6.7%)	34 (9.1%)	0.342
Tachycardia	33 (6.0%)	17 (9.6%)	16 (4.3%)	0.015
Headache	26 (4.7%)	7 (3.9%)	19 (5.1%)	0.544
Dizziness	18 (3.3%)	4 (2.2%)	14 (3.8%)	0.350
Sneeze	1 (0.2%)	0 (0.0%)	1 (0.3%)	1.000
Arthralgia	1 (0.2%)	0 (0.0%)	1 (0.3%)	1.000
Multiple symptoms	393 (71.5%)	162 (91.0%)	231 (62.1%)	< 0.001
Fever, cough^a^, and dyspnea	130 (23.6%)	76 (42.7%)	54 (14.5%)	< 0.001
Radiographic findings^b^, n (%)				
Bilateral pneumonia	378/482 (78.4%)	146/161 (90.7%)	232/321 (72.3%)	< 0.001
Unilateral pneumonia	50/482 (10.4%)	5/161 (3.1%)	45/321 (14.0%)	< 0.001
Normal	7/482 (1.5%)	0/161 (0%)	7/321 (2.2%)	< 0.001
Others	47/482 (9.7%)	10/161 (6.2%)	37/321 (11.5%)	< 0.001
Etiological findings, n (%)				
Phlegm smear				
Gram-positive bacilli	3/34 (8.8%)	1/11 (9.1%)	2/23 (8.7%)	0.374
Gram-negative bacilli	12/34 (35.4%)	3/11 (27.3%)	9/23 (39.1%)	0.374
Cocci	10/34 (29.4%)	2/11 (18.2%)	8/23 (34.8%)	0.374
Fungus	1/34 (2.9%)	1/11 (9.1%)	0/23 (0.0%)	0.374
Normal	8/34 (23.5%)	4/11 (36.3%)	4/23 (17.4)	0.374
Mycoplasma/Chlamydia Pneumoniae antibody (IgM)				
Positive	27/350 (7.7%)	4/121 (3.3%)	23/229 (10.0%)	0.042
Negative	323/350 (92.3%)	117/121 (96.7%)	206/229 (90.0%)	0.042
Respiratory pathogen antibody				
Positive	29/342 (8.5%)	13/123 (10.6%)	16/219 (7.3%)	0.299
Negative	313/342 (91.5%)	110/123 (89.4%)	203/219 (92.7%)	0.299
CMV/EBV				
Positive	10/154 (6.5%)	4/54 (7.4%)	6/100 (6.0%)	1.000
Negative	144/154 (93.5%)	50/54 (92.6%)	94/100 (94.0%)	1.000
Influenza Virus Antigen				
Positive	3/265 (1.1%)	1/103 (1.0%)	2/162 (1.2%)	1.000
Negative	262/265 (98.9%)	102/103 (99.0%)	160/162 (98.8%)	1.000
Admission	9 (6–14)	10 (7–12)	9 (5.75–15)	0.797
Dyspnea	0 (0–7)	2 (0–8)	0 (0–6)	0.007
Mechanical ventilation	10 (6–15)	10 (6.75–15)	–	–
ARDS	10 (6–15)	10 (6.75–15)	–	–

Data are shown as median (IQR) or n (%). *ARDS* Acute respiratory distress syndrome, *CMV* Cytomegalovirus. *EBV* Epstein-Barr virus

^a^Cough includes dry cough and expectoration

^b^Radiographic findings include the findings of both the chest X-ray and lung CT scan. When “viral pneumonia” was reported only, without a description of the lesion sites, the results were marked by others

The median time from disease onset to admission was 9 (IQR, 6–14) days, the time from disease onset to dyspnea was 0 (IQR, 0–7) days. In this cohort, 69 patients eventually developed ARDS and needed mechanical ventilation. The average time from disease onset to ARDS was 10 (IQR, 6–15) days among patients who eventually developed ARDS, and the mean time to mechanical ventilation was 10 (IQR, 6–15) days in the same subgroup.

Compared with the patients who did not progress to a severe condition, the severe patients were generally older and had a higher proportion of males (*n* = 104, 58.4% vs. 41.4%, *P* < 0.001). Patients with clear exposure histories were more often non-severe (*P* < 0.001), while the exposure history was not traceable in most severe patients (*P* < 0.001). For clinical symptoms, most of the non-severe patients did not have a fever upon hospital admission (*n* = 157, 42.3%, *P* < 0.001). Patients presenting with moderate or severe fever were more likely to have disease progression (*P* < 0.001), defined as when the illness turned to more severe or critical conditions or death. In addition, dyspnea, fatigue, chill, sputum production, and tachycardia were more common in severe patients. Severe patients were frequently associated with multiple clinical symptoms, especially the classic triple signs (*n* = 76, 42.7%, *P* < 0.001) (Table 2).

### Laboratory and imaging findings

Upon hospital admission, all patients underwent relevant laboratory examinations in order to assess the patients’ condition and guide treatments (Table 3). The results indicated that 23.7% of the patients (119/502) had leukopenia, which was more frequently seen in severe patients (*P* < 0.001). In patients with lymphocyte count < 1.0 × 10^9^/L, 130/169 (76.9%) patients eventually developed severe disease. The levels of other inflammatory indicators such as procalcitonin (PCT), highly-sensitive C-reactive protein (hsCRP), and erythrocyte sedimentation rate (ESR) were increased in severe patients compared to non-severe patients (*P* < 0.001). In addition, myocardial enzymes were elevated in severe patients, and 85/128 (66.4%) of severe patients presented elevated NT-proBNP levels (*P* < 0.001). Elevation of alanine and aspartate aminotransferase occurred more frequently in severe patients, and 163/175 (93.0%) severe patients had hypoproteinemia (*P* < 0.001). A relatively small number of patients developed a reduced glomerular filtration rate, but it was more commonly seen in severe patients (24/176, 13.6%, *P* < 0.001). Furthermore, more patients in the severe group (108/133, 81.2%) had elevated D-dimer levels compared to non-severe patients. Moreover, severe patients were more likely to be associated with electrolyte disorders. Blood gas analysis revealed that 75.2% (124/165) of severe patients had an oxygen index (OI) < 300 at admission, of which 26 patients had OI < 100. There was no difference in the proportion of patients with hyperlactatemia between the two groups (*P* = 0.172).

**Table 3:** Laboratory results of patients with COVID-19 on hospital admission

Variables	All Patients (*n* = 550)	Severe (*n* = 178)	Non-severe (*n* = 372)	*p* value
Blood tests, n/total n (%)				
Leucocytes (×10^9^/L)				
< 4	119/502(23.7%)	21/169(12.4%)	98/333(29.4%)	< 0.001
4–10	335/502(66.7%)	110/169(65.1%)	225/333(67.6%)	< 0.001
> 10	48/502(9.6%)	38/169(22.5%)	10/333(3.0%)	< 0.001
Neutrophil percentage (%)				
40–75	321/502(63.9%)	47/169(27.8%)	274/333(82.3%)	< 0.001
> 75	175/502(34.9%)	122/169(72.2%)	53/333(15.9%)	< 0.001
Lymphocyte percentage (%)				
< 20	230/502(45.8%)	143/169(84.6%)	87/333(26.1%)	< 0.001
20–50	267/502(53.2%)	26/169(15.4%)	241/333(72.4%)	< 0.001
Lymphocytes (×10^9^/L)				
< 1.0	250/502(49.8%)	130/169(76.9%)	120/333(36.0%)	< 0.001
≥ 1.0	252/502(50.2%)	39/169(23.1%)	213/333(64.0%)	< 0.001
Hemoglobin (g/L)				
Normal	317/502(63.1%)	108/169(63.9%)	209/333(62.8%)	0.802
Decreased	185/502(36.9%)	61/169(36.1%)	124/333(37.2%)	0.802
Platelets (×10^9^/L)				
< 100	34/502(6.8%)	13/169(7.7%)	21/333(6.3%)	0.559
≥ 100	468/502(93.2%)	156/169(92.3%)	312/333(93.7%)	0.559
Inflammatory parameters-no./total no. (%)				
Procalcitonin (ng/ml)				
≤ 0.1	279/393(71.0%)	63/149(42.3%)	216/244(88.5%)	< 0.001
> 0.1	117/393(29.0%)	86/149(57.7%)	28/244(11.5%)	< 0.001
hsCRP (mg/L)				
≤ 3	120/404(29.7%)	5/136(3.7%)	115/268(42.9%)	< 0.001
> 3	284/404(70.3%)	131/136(96.3%)	153/268(57.1%)	< 0.001
ESR (mm/h)				
≤ 15	74/185(40.0%)	3/55(5.5%)	71/130(54.6%)	< 0.001
> 15	111/185(60.0%)	52/55(94.5%)	59/130(45.4%)	< 0.001
Myocardial enzyme-no./total no. (%)				
CK-MB (ng/mL)				
≤ 6.22	399/422(94.5%)	130/146(89.0%)	269/276(97.5%)	< 0.001
> 6.22	23/422(5.5%)	16/146(11.0%)	7/276(2.5%)	< 0.001
Troponin T (ng/ml)				
≤ 0.014	331/438(75.6%)	82/161(50.9%)	249/277(89.9%)	< 0.001
> 0.014	107/438(24.4%)	79/161(49.1%)	28/277(10.1%)	< 0.001
Heart Failure Indicator-no./total no. (%)				
BNP (pg/ml)				
≤ 222	166/291(57.0%)	43/128(33.6%)	123/163(75.5%)	< 0.001
> 222	125/291(43.0%)	85/128(66.4%)	40/163(24.5%)	< 0.001
Liver function-no./total no. (%)				
Alanine transaminase (IU/L)				
≤ 50	443/511(86.7%)	136/175(77.7%)	307/336(91.4%)	< 0.001
> 50	68/511(13.3%)	39/175(22.3%)	29/336(8.6%)	< 0.001
Aspartate aminotransferase (IU/L)				
≤ 40	393/511(76.9%)	95/175(54.3%)	298/336(88.7%)	< 0.001
> 40	118/511(23.1%)	80/175(45.7%)	38/336(11.3%)	< 0.001
Albumin (g/L)				
< 40	345/511(67.5%)	163/175(93.1%)	182/336(54.2%)	< 0.001
40–55	166/511(32.5%)	12/175(6.9%)	154/336(45.8%)	< 0.001
Coagulation Function, n/total n (%)				
APTT (S)				
24.6–35.4	364/430(84.7%)	136/158(86.1%)	228/272(83.8%)	0.532
> 35.4	66/430(15.3%)	22/158(13.9%)	44/272(16.2%)	0.532
D-dimer (μg/ml)				
≤ 0.243	182/364(50.0%)	25/133(18.8%)	157/231(68.0%)	< 0.001
> 0.243	182/364(50.0%)	108/133(81.2%)	74/231(32.0%)	< 0.001
Electrolyte, n/total n (%)				
Potassium (mmol/L)				
> 5.3	39/504(7.7%)	22/172(12.8%)	17/332(5.1%)	< 0.001
3.5–5.3	389/504(77.2%)	109/172(63.4%)	280/332(84.4%)	< 0.001
< 3.5	76/504(15.1%)	41/172(23.8%)	35/332(10.5%)	< 0.001
Sodium (mmol/L)				
< 137	72/504(14.3%)	44/172(25.6%)	28/332(8.4%)	< 0.001
137–147	411/504(81.5%)	115/172(66.8%)	296/332(89.2%)	< 0.001
> 147	21/504(4.2%)	13/172(7.6%)	8/332(2.4%)	< 0.001
Renal Function, n/total n (%)				
Creatinine (μmol/L)				
≤ 111	480/508(94.5%)	160/176(90.9%)	320/332(96.4%)	0.010
> 111	28/508(5.5%)	16/176(9.1%)	12/332(3.6%)	0.010
GFR				
< 66	36/508(7.1%)	24/176(13.6%)	12/332(3.6%)	< 0.001
≥ 66	472/508(92.9%)	152/176(86.4%)	320/332(96.4%)	< 0.001
Arterial blood gas analysis, n/total n (%)				
PH				
< 7.35	23/338(6.8%)	14/165(8.5%)	9/173(5.2%)	< 0.001
7.35–7.45	233/338(68.9%)	90/165(54.5%)	143/173(82.7%)	< 0.001
> 7.45	82/338(24.3%)	61/165(37.0%)	21/173(12.1%)	< 0.001
Oxygenation index				
< 100	26/338(7.7%)	26/165(15.8%)	0/173(0.0%)	< 0.001
100–300	98/338(29.0%)	98/165(59.4%)	0/173(0.0%)	< 0.001
> 300	214/338(63.3%)	41/165(24.8%)	173/173(100.0%)	< 0.001
PCO2 (mmHg)				
< 35	73/338(21.6%)	52/165(31.5%)	21/173(12.1%)	< 0.001
35–45	185/338(54.7%)	90/165(54.6%)	95/173(55.0%)	< 0.001
> 45	80/338(23.7%)	23/165(13.9%)	57/173(32.9%)	< 0.001
Lactic acid (mmol/L)				
≤ 2.2	245/338(72.5%)	114/165(69.1%)	131/173(75.7%)	0.172
> 2.2	93/338(27.5%)	51/165(30.9%)	42/173(24.3%)	0.172

The data were expressed in the form of n/N (%), where N represents the total number of patients with available data

*hsCRP* Hypersensitive c-reactive protein, *ESR* Erythrocyte sedimentation rate, *CKMB* Creatine kinase isoenzyme, *BNP* B-type natriuretic peptide, *APTT* Activated partial thromboplastin time, *GFR* Glomerular filtration rate, *PCO2* Partial pressure of carbon dioxide

**Fig. 1:**
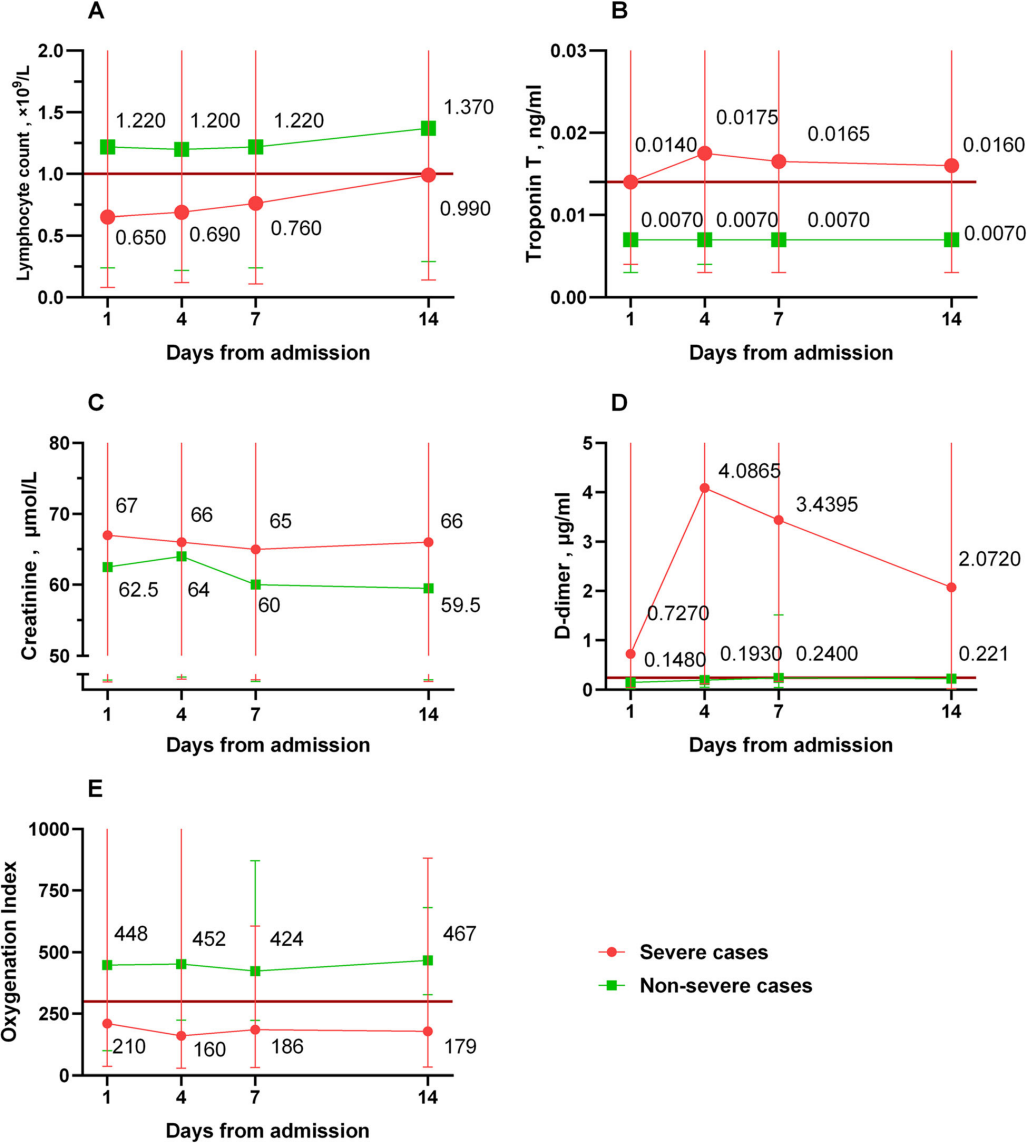
Changes in laboratory parameters in patients with COVID-19 infection. The changes in lymphocyte counts (**A**), troponin T (**B**), creatinine (**C**), D-dimer (**D**), and oxygenation index (**E**) were recorded. The differences between severe and non-severe cases were statistically significant at all time points except for creatinine on the 4th, 7th, and 14th days after admission (*P* < 0.05). The normal values of the parameters are shown as the red solid line. COVID-19 = 2019 novel coronavirus disease

Lymphocyte count, troponin T, serum creatinine, D-dimer level, and OI were closely monitored and compared between the severe and non-severe groups (Fig. 1). The lymphocyte counts were lower in severe patients but increased more robustly after day 7 compared with non-severe patients. Troponin T and D-dimer levels were higher in severe patients and peaked around the 4th day after admission. There was no significant difference in creatinine levels between the two groups except on 1st day of admission. In addition, severe hypoxemia was more common in severe patients.

In this cohort, only a very small number of patients were co-infected with other pathogens such as bacteria, influenza virus, and atypical pathogens (Table 2). Among all patients, 482 patients (87.6%) had completed chest radiographs or lung CT scans during hospitalization. For all 161 patients that progressed into advanced stages with radiologic assessments, 146 (90.7%) had bilateral lung lesions. Only 72.3% (232/321) of the non-severe patients developed bilateral lung lesions (Table 2).

### Complications

The most common complications were acute myocardial injury (*n* = 111, 20.2%), secondary infection (*n* = 110, 20.0%), ARDS (*n* = 69, 12.5%), acute renal injury (*n* = 45, 8.2%), shock (*n* = 40, 7.3%), and disseminated intravascular coagulation (DIC) (*n* = 20, 3.6%). Unsurprisingly, severe patients were more likely to develop complications (Table 4).

**Table 4:** Complications, treatment, and prognosis of patients with COVID-19

Variables	All Patients (*n* = 550)	Severe (*n* = 178)	Non-severe (*n* = 372)	*p* value
Complications n, %				
Acute myocardial injury	111(20.2%)	76(42.7%)	35(9.4%)	< 0.001
Secondary infection	110 (20.0%)	82(46.1%)	28(7.5%)	< 0.001
ARDS	69 (12.5%)	69(38.8%)	0	< 0.001
Acute kidney injury	45 (8.2%)	33(18.5%)	12(3.2%)	< 0.001
Shock	40 (7.3%)	39(21.9%)	1(0.3%)	< 0.001
DIC	20(3.6%)	11(6.2%)	9(2.4%)	0.028
Treatment n.%				
Antiviral therapy	449 (81.6%)	162(91.0%)	287(77.2%)	< 0.001
Antibacterial therapy				
one kind	204 (37.1%)	40(22.5%)	164(44.1%)	< 0.001
≥ two kinds	231(42.0%)	136(76.4%)	95(25.5%)	< 0.001
Antifungal therapy	10(1.8%)	8(4.5%)	2(0.5%)	0.004
Glucocorticoids therapy	191(34.7%)	122(68.5%)	69(18.5%)	< 0.001
Immunotherapy				
Human immunoglobulin	52(9.5%)	23(12.9%)	29(7.8%)	0.055
Thymosin	10(1.8%)	6(3.4%)	4(1.1%)	0.123
Vasoactive drug	34(6.2%)	34(19.1%)	0	< 0.001
CRRT	2(0.4%)	2(1.1%)	0	0.104
Respiratory support-no.%				
Nasal catheter/Mask oxygen	477(86.8%)	105(59.0%)	372(100.0%)	< 0.001
High-flow nasal cannula	5 (0.9%)	5(2.8%)	0	< 0.001
Noninvasive ventilation	32 (5.8%)	32(18.0%)	0	< 0.001
Invasive ventilation	36 (6.5%)	36(20.2%)	0	< 0.001
ECMO	0	0	0	–
prognosis-no.%				
Transfer	24 (4.4%)	16 (9.0%)	8 (2.2%)	< 0.001
Improved	474 (86.1%)	110 (61.8%)	364 (97.8%)	< 0.001
Death	52(9.5%)	52(29.2%)	0	< 0.001
Multiple system and organ failure	33/52 (63.5%)	33/52 (63.5%)	–	–
Respiratory failure	16/52 (30.8%)	16/52 (30.8%)	–	–
Circulatory failure	2/52 (3.8%)	2/52 (3.8%)	–	–
Septic shock	1/52 (1.9%)	1/52 (1.9%)	–	–
Negative conversion time of RNA Detection, Median (IQR)-days	10 (6–16)	13 (8–18)	9 (6–16)	0.016
Length of hospital stay, Median (IQR)-days	16 (9–26)	22 (13–30)	15 (9–22.75)	< 0.001

### Identification of risk factors for severe cases

The multivariable logistic regression analysis showed that age ≥ 60 years (OR = 3.02, 95%CI: 1.13–8.08, *P* = 0.028) and D-dimer > 0.243 μg/ml (OR = 2.73, 95%CI: 1.01–7.39, *P* = 0.047) were independently associated with severe cases (Table 5). A decrease in OI (OR = 0.984, 95%CI: 0.980–0.999, *P* < 0.001) was also independently associated with disease deterioration.

**Table 5:** Early warning indicators for the occurrence of severe cases with COVID-19

	Univariable OR (95%CI)	*p* value	Multivariable OR (95%CI)	*p* value
Demographics and clinical characteristics				
Age, years				
≥ 60	3.985 (2.701–5.879)	< 0.001	3.022 (1.130–8.083)	0.028
Sex				
Male	2.034 (1.415–2.924)	< 0.001		
Comorbidity				
Hypertension	2.152 (1.484–3.121)	< 0.001	0.724 (0.263–1.999)	0.531
Diabetes	2.178 (1.336–3.550)	0.002		
Heart disease	2.023 (1.152–3.552)	0.014		
Cancer	2.155 (0.88–5.276)	0.093		
Temperature, °C				
≥ 38.0	2.010 (1.103–3.663)	0.023	1.355 (0.536–3.423)	0.521
Symptom				
More than one sign or symptom	2.841 (1.941–4.157)	< 0.001		
Fever, cough and dyspnea	2.373 (1.580–3.566)	< 0.001		
Radiographic and laboratory findings				
Radiographic findings ^a^				
Bilateral pneumonia	6.419 (2.253–18.287)	< 0.001		
Leucocytes (×10^9^/L)				
< 4	0.119 (0.053–0.267)	< 0.001		
> 10	0.056 (0.023–0.138)	< 0.001		
Lymphocyte count (×10^9^/L)				
LN < 1.0	3.297 (2.213–4.912)	< 0.001	1.903 (0.736–4.923)	0.184
Procalcitonin (ng/ml)				
> 0.1	6.860 (4.222–11.146)	< 0.001		
Troponin T (ng/ml)				
> 0.014	9.465 (5.412–16.554)	< 0.001		
D-dimer (μg/ml)				
> 0.243	4.375 (2.191–8.734)	< 0.001	2.734 (1.012–7.387)	0.047
Glomerular filtration rate				
< 66	4.375 (2.191–8.734)	< 0.001		
Oxygenation index	0.986 (0.983–0.989)	< 0.001	0.984 (0.980–0.989)	< 0.001
Lactic acid (mmol/L)				
> 2.2	1.547 (0.939–2.551)	0.087		

Univariable and multivariable logistic regression analyses were performed, and six variables were selected for further multivariable. *OR* Odds ratio

^a^ Radiographic findings include the findings of both chest X-ray and lung CT scan

### Treatments

All patients (100.0%) were given intermittent or continuous oxygen inhalation therapy to improve the clinical symptoms (Table 4). Among the severe patients, 20.2% required invasive ventilator support, 18.0% required non-invasive ventilator, and 2.8% required high-flow nasal cannula, while the remaining patients were treated with nasal catheters/masks for oxygen therapy. No ECMO was used.

Among the patients, 79.1% were treated with antibiotics, and 231 (42.0%) were treated with more than one type of antibiotics. The choice of antibiotics was based on the local epidemiological situation in Wuhan. Most of the patients with severe diseases were treated with a combination of moxifloxacin and cefoperazone /sulbactam. The critical patients are often treated with imipenem or biapenem. Non-critical patients often received moxifloxacin, combined cefoperazone/sulbactam, or azithromycin. The most frequently used drugs were moxifloxacin (*n* = 407, 74.0%), cephalosporins/ sulbactam (*n* = 186, 33.8%), carbapenems (*n* = 61, 11.1%), and azithromycin (*n* = 52, 9.5%). A higher percentage of patients in the severe group received intravenous or oral glucocorticoids compared with the non-severe patients (122/178, 68.5% vs. 69/372, 18.5%, *P* < 0.001).

Among all patients, 81.6% were treated with antiviral drugs, and the remaining 18.4% were treated only with traditional Chinese medicine. The outcomes of the patients treated with antiviral drugs are shown in Table 6. The antiviral drugs used in this study were arbidol (*n* = 240, 43.6%), oseltamivir (*n* = 216, 39.3%), ribavirin (*n* = 152, 27.6%), lopinavir/ritonavir (*n* = 21, 3.8%), and α-interferon (*n* = 20, 3.6%). Arbidol was more effective than ribavirin (73.3% vs. 41.2%, *P* = 0.029) in treating severe patients as single-drug therapy when considering symptom relief, clinical outcome, and discharge rate. Similarly, in severe patients who were treated with two drugs, arbidol combined with ribavirin or oseltamivir also had better efficacy. There were no significant differences identified among the other treatments. Some patients also received immunotherapies, including human immunoglobulin infusion (*n* = 52, 9.5%) and thymosin (*n* = 10, 1.8%). Vasoactive drugs were used in 34 severe cases, and continuous blood purification therapy was used in two cases.

**Table 6:** Antiviral efficacy in patients with COVID-19

	Severe (*n* = 178)		Non-severe (*n* = 372)	
Ribavirin	7/17	41.2%	22/23	95.7%
Oseltamivir	34/53	64.2%	49/49	100.0%
Arbidol	22/30	73.3%	109/110	99.1%
Lopinavir/ritonavir	0/0	–	2/2	100%
Ribavirin+Oseltamivir	9/20	45.0%	38/38	100%
Ribavirin+Arbidol	12/12	100%	18/19	94.7%
Arbidol+Oseltamivir	15/17	88.2%	14/16	87.5%

The data were expressed in the form of n/N (%), where n represents the number of patients with clinical disease improvement, N represents the total number of patients receiving corresponding drugs

### Patient outcomes

After treatment, 474 (86.1%) patients’ conditions were improved, 24 (4.4%) patients were transferred to superior hospitals, and 52 (9.5%) patients passed away due to multiple organ failure (63.5%), respiratory failure (30.8%), circulatory failure (3.8%), and septic shock (1.9%). Next, the MuLBSTA scoring system was used to score the mortality cases and showed that 46 patients belonged to the high-risk group of death, with a median score of 17 (15–17), while six cases were in the low-risk group, with a median score of 9 (8.25–10.5). The median hospitalization time was 16 (IQR, 9–26) days for all patients and 22 (IQR, 13–30) days for severe patients.

## Discussion

Because the incubation period of COVID-19 can be up to 3 weeks [1–4], the early identification of patients at higher risk of severe disease is important to implement timely medical strategies. This could save time and energy in the context of the exhausted healthcare systems. The results suggest that age ≥ 60 years, D-dimer > 0.243 μg/ml, and lower oxygenation index were associated with severe COVID-19. Therefore, the patients presenting those characteristics could be more aggressively managed from the start in order to prevent complications. In addition, arbidol might provide more clinical benefits in treating patients with severe COVID-19 compared with other antiviral drugs.

A total of 550 patients diagnosed with COVID-19 were included in this study. Inconsistent with the literature, there were more females (53.1%) than males in the present study [1–4], but there were more male patients among severe cases. This discrepancy can be due to many factors, including the transmission route, willingness to undergo screening, and socioeconomic factors. Epidemiology tracing identified 170 (30.9%) of patients with 2019-nCoV having a history of contact with an infected individual and 38 (6.9%) due to a recent visit to a COVID-19-designated hospital. The remaining of the patients had no clear source of infection. Those results highlight the need for refraining from having contacts and from enforcing physical distancing, from avoiding visiting hospitals known to treat COVID-19 and visiting other hospitals, and that many patients might have been infected through asymptomatic, either because those patients were asymptomatic carriers or because symptom onset did not occur yet. This will have to be examined in future studies.

In this study, 42.3% of the non-severe patients did not have a fever at diagnosis, which was lower than what was reported by Guan et al. [4]. Upon hospital admission, 42.7% of the patients who eventually progressed to severe COVID-19 had the typical triple signs (fever, cough, and dyspnea), while the triple signs were observed in only 14.5% of the non-severe patients. Unsurprisingly, severe patients often had more comorbidities. Hypertension, diabetes, cardiovascular diseases, and malignancy were the most common underlying diseases observed in patients with severe COVID-19. Older age was also observed to be associated with severe COVID-19, which is consistent with recent studies [4, 28], but whether this is because older patients can be frailer and weaker or because older individuals often have more comorbidities is still unknown.

As for the laboratory tests, 66.7% of the patients in the study had normal leukocyte count, and a quarter of the patients had decreased WBC counts. For 9.6% of patients who had an elevated WBC, secondary infections were often the cause of the elevated WBC. In addition, 49.8% of the patients presented with decreased lymphocyte counts, of which 52.0% (130/250) were severe cases. The incidence of anemia and thrombocytopenia was 36.9 and 6.8%, respectively, without differences according to disease severity. Cardiac enzymes, troponin T, transaminase, creatinine, and other organ injury indicators were also increased to varying degrees in some patients. Both hsCRP and ESR were increased in most patients, especially in severe patients. This highlights the systemic nature of the disease and that the patients should be comprehensively assessed. The increased inflammatory indicators suggested that SARS-CoV-2 tips the balance of the immune system towards a cytokine storm that contributes to patient deterioration and mortality, as observed in various infections [29, 30]. Recent biopsy reports by Xu et al. [31] also indicated an increase of proinflammatory CCR4+ CCR6+ Th17 cells in the peripheral blood that might lead to systemic inflammatory responses and contribute to diffuse alveolar injury and pulmonary hyaline membrane formation. That evidence suggests that the systemic inflammatory response is an important factor leading to poor COVID-19 prognosis, as supported by the literature [29, 30]. Unfortunately, due to the limited conditions of the hospital, no cytokine or other related testing was performed in this study. Future studies should aim to carefully examine the various cytokines involved in COVID-19 and in relation to disease severity.

It has been estimated that the mortality rate in severe cases was over 50% [32]. Therefore, it is critical to identify patients with an increased risk of disease progression. In the present study, the multivariable logistic regression analysis showed that age ≥ 60 years, D-dimer > 0.243 μg/ml, and decreased OI might be risk factors for patient deterioration. The importance of aging in determining the COVID-19 prognosis was consistent with previous studies that aimed to identify prognosis factors for SARS or MERS infections [28, 33–35]. The coagulation dysfunction we observed in this study was consistent with previous studies [1–3, 5]. Severe patients were more likely to develop coagulation and fibrinolysis disorders, especially the elevation of D-dimer levels. Similar to other severe viral pneumonia, the cause might be the sepsis-induced inflammatory cytokine storm affecting multiple endogenous and exogenous coagulation pathways and fibrinolysis that ultimately lead to thrombosis formation [17, 29]. Therefore, special attention should be paid to severe patients with long-term bed rest, advanced age, and complicated underlying diseases, especially in the presence of coagulation abnormalities. Appropriate anticoagulant treatment might be considered in such patients in order to prevent the occurrence of deep vein thrombosis (DVT) and related complications [36–38]. In the present study, a reduction in OI was associated with increased mortality. Similarly, Liu et al. [39] found that the lung injury Murray score and OI could predict the prognosis of COVID-19. Therefore, early recognition of these three indicators upon hospital admission is critical, so appropriate medical strategies can be adjusted, and more importantly, the nearly exhausted medical support force can be redistributed. This is especially important because when the patients are admitted, the exact interval between infection and symptom onset is unknown, and the exact time until an eventual disease progression is also unknown.

In this study, 435 patients (79.1%) received at least one antibiotic in the hospital, but only 110 (20.0%) of them were confirmed with secondary bacterial infection (some cases were accompanied by fungal infection). Therefore, more attention should be paid to the indication of antibiotic use and avoid antibiotic overuse. Prophylaxis for eventual complicating secondary bacterial or fungal infections can be indicated in some cases, but additional studies are necessary to determine who they might be. Since patients with severe and critical COVID-19 have a compromised immune system [40, 41], the rationale for prophylactic antibiotics is to avoid a secondary infection that might worsen the condition of the patients and his prognosis. In addition, 122 severe patients received intravenous or oral glucocorticoid treatment. Among these patients, 79 had an improved condition, but 43 eventually died. Nevertheless, the rate of improvement was relatively high (83.9% vs. 64.8%, *P* = 0.009) in severe patients who did not receive glucocorticoids. It is important to point out that the patients who received glucocorticoids also had a higher rate of secondary infections compared with patients who did not receive glucocorticoids (36.9% vs. 19.6%, *P* = 0.021). This is consistent with several recent studies that suggested that glucocorticoids are not beneficial for patients with viral infections [42, 43], but contradicts recent findings that suggest that corticosteroids decrease the mortality of COVID-19, but the level of evidence is low [44]. A study in SARs showed that early glucocorticoids increased the viral load [45], and another study in MERS reported delayed virus clearance [46]. Even though this study was not powered to analyze the benefit and risks of using glucocorticoids in COVID-19, the data suggest that glucocorticoids failed to improve the prognosis and increased the risk of secondary infection.

Another important feature of this study was the assessment of current antiviral drugs. The antiviral drugs used during the study period were arbidol, oseltamivir, ribavirin, lopinavir/ritonavir, and α- interferon. The results suggest that arbidol might provide more benefits compared with ribavirin in severe patients treated with monotherapy, but the difference between arbidol and oseltamivir was not significant (*P* = 0.391). At the same time, in severe patients who received combination therapy, the combinations that included arbidol showed better benefits. A multicenter randomized controlled clinical trial published on medRxiv recently showed that patients treated with favipiravir had a better recovery rate (71.4% vs. 55.9% *P* = 0.0199) but more side effects were observed compared with arbidol [16, 18]. There are multiple antiviral drugs being evaluated and tested in trials currently [19], but before better options can be justified, the use of arbidol might be recommended for its relative safety and effectiveness profile.

Using the MuLBSTA scoring system, 46 (88.5%) patients were correctly classified as at high-risk for death (score > 12), but only 22 (42.3%) were correctly classified as high-risk (score ≥ 2) when using the CURB-65 scoring system. This suggests that the MuLBSTA scoring system is more effective in the mortality risk assessment of patients with COVID-19 (*P* < 0.001) in the early stage of the disease. This is consistent with a previous study [47]. We speculated that the reason why the MuLBSTA scoring system was more effective is that its scoring criteria (age ≥ 60, smoking status/smoking cessation history, hypertension history, imaging showing multiple lobar infiltrations, lymphocyte counts ≤0.8 × 10^9^/L, or combined with a bacterial infection) can be achieved and evaluated at the early stage of the disease. On the other hand, the parameters analyzed in CURB-65 may not be elevated in the early stage of the disease in the high-risk population. If necessary, an attempt might be made to lower the scoring criteria and set 1 as a cut-off point to improve its sensitivity (44/52, 84.6% vs. 46/52, 88.5%, *P* = 0.566). Despite that the 2009 IDSA/ATS guidelines recommended CURB-65 as a severity assessment form for community-acquired pneumonia (CAP) [48], the present study suggests that the MuLBSTA scoring system might be a better assessment tool for COVID-19.

### Limitations

This study has several limitations. First, this was a retrospective study conducted at a single center, with a cohort of 550 patients treated after the arrival of the Hebei medical team, which might not necessarily represent the general population of patients. In addition, the false-negative rates of current SARS-CoV-19 tests are relatively high and might bias the results. Last but not least, due to the retrospective nature of this study and the lack of diverse drugs in the early stage of the epidemic, the observation of the benefits for different antiviral drugs needs to be further confirmed in future randomized controlled trials.

## Conclusion

In conclusion, age ≥ 60 years, D-dimer > 0.243 μg/ml, and lower OI could help clinicians identify patients with increased probabilities for disease progression early and implement timely medical strategies. Arbidol might have benefits in treating severe patients, but there was only one comparator drug in this study (ribavirin), but the efficacy and safety of drugs for COVID-19 still need to be assessed in future clinical trials.

## Data Availability

The datasets used and/or analyzed during the current study are available from the corresponding author on reasonable request.

## Abbreviations

ARDS: Acute respiratory distress syndrome
MODS: Multiple organ failure
WHO: World Health Organization
CT: Computed tomography
IQR: Interquartile range
OI: Oxygen index
CAP: Community-acquired pneumonia
